# The Glycosylphosphatidylinositol-PLC in *Trypanosoma brucei* Forms a Linear Array on the Exterior of the Flagellar Membrane Before and After Activation

**DOI:** 10.1371/journal.ppat.1000468

**Published:** 2009-06-05

**Authors:** Orla Hanrahan, Helena Webb, Robert O'Byrne, Elaine Brabazon, Achim Treumann, Jack D. Sunter, Mark Carrington, H. Paul Voorheis

**Affiliations:** 1 School of Biochemistry and Immunology, Trinity College Dublin, Dublin, Ireland; 2 Department of Biochemistry, University of Cambridge, Cambridge, United Kingdom; 3 Royal College of Surgeons in Ireland, Dublin, Ireland; Yale University, United States of America

## Abstract

Bloodstream forms of *Trypanosoma brucei* contain a glycosylphosphatidylinositol-specific phospholipase C (GPI-PLC) that cleaves the GPI-anchor of the variable surface glycoprotein (VSG). Its location in trypanosomes has been controversial. Here, using confocal microscopy and surface labelling techniques, we show that the GPI-PLC is located exclusively in a linear array on the outside of the flagellar membrane, close to the flagellar attachment zone, but does not co-localize with the flagellar attachment zone protein, FAZ1. Consequently, the GPI-PLC and the VSG occupy the same plasma membrane leaflet, which resolves the topological problem associated with the cleavage reaction if the VSG and the GPI-PLC were on opposite sides of the membrane. The exterior location requires the enzyme to be tightly regulated to prevent VSG release under basal conditions. During stimulated VSG release in intact cells, the GPI-PLC did not change location, suggesting that the release mechanism involves lateral diffusion of the VSG in the plane of the membrane to the fixed position of the GPI-PLC.

## Author Summary

African trypanosomes cause sleeping sickness, for which current therapy is inadequate. The parasite protects its surface from the host immune system by regularly switching its surface coat. The glycosylphosphatidylinositol-PLC only occurs in the bloodstream form, where it removes the surface coat after it enters the tsetse fly vector. Activation of the enzyme in the bloodstream would be fatal for the parasite and it is, therefore, a potential drug target. However, therapeutic strategies have been hampered by confusion over the location of the GPI-PLC despite great effort by many labs. We have used a wide variety of techniques, including one completely novel method, that exploits the dependence of detection for partially buried surface proteins on the temperature of fixation, to identify the location of the GPI-PLC in relation to other markers unequivocally. All approaches consistently show that the GPI-PLC is located exclusively in the outer leaflet of the plasma membrane covering the flagellum, where it is confined to a narrow linear array adjacent to the flagellar attachment zone. Our data have resolved the question of how enzyme and substrate meet and also suggest that chemotherapeutic agents would be able to target the GPI-PLC in its exterior location.

## Introduction

The GPI-PLC is present in bloodstream forms of *T. brucei* [1],[2],[3] and in many other eukaryotic cells. The enzyme cleaves the GPI-anchor of the VSG and other GPI-anchored proteins, provided the 2-position on the inositol is underivatized, forming free diacylglycerol in the membrane and, probably, a 1,2-cyclic phosphate on the inositol ring, which remains attached to the released VSG [4],[5]. This cleavage converts the membrane-bound form of the VSG (mVSG) to the soluble released form of the VSG (sVSG) [6]. The conversion can be detected immunologically because it exposes the cross-reacting determinant (CRD), which contains the inositol-1,2-cyclic phosphate [6],[7].

VSG release can be initiated by calcium ion influx into intact trypanosomes [8],[9] hypotonic lysis of cells, [6],[10],[11] and by acidic stress in long slender bloodstream forms [12],[13]. These observations suggested that VSG release is a regulated process involving calcium ions. Importantly, catalytic activity itself does not require Ca^2+^ [1],[3].

The GPI-PLC is only present in metacyclic and bloodstream stages of the life cycle where the parasite is covered with a VSG coat [14],[15],[16]. Procyclic forms do not contain GPI-PLC enzymatic activity [14],[17],[18]. The normal cellular functions of the GPI-PLC in vivo are not completely clear. However, deletion of both the genes encoding the GPI-PLC and a Zn^2+^-metalloprotease prevents the proliferation and differentiation of bloodstream forms of *T. brucei* into procyclic forms [19]. It would be helpful to know the exact location of the GPI-PLC under basal, unstimulated as well as stimulated conditions in order to confirm this function of the GPI-PLC by a different approach, to determine any other functions of the GPI-PLC and to investigate the regulation of the enzyme. This knowledge would be particularly helpful in a cell as polarized as *T. brucei*, where functions can be very localized, even within the same membrane. However, considerable controversy exists over the location of the GPI-PLC. In fact the following different locations for the GPI-PLC have been reported:

1. The plasma membrane [20]
2. In *or* enclosed by membranes of the flagellum, cell body and Golgi [14]
3. Cytoplasmic face of intracellular vesicles located between the Golgi and the flagellar pocket [21]
4. Internal leaflet of the plasma membrane in intact cells *but* migrating around the edge of the ruptured membrane in broken cells to reside, finally, in the external leaflet of the plasma membrane [22]
5. External surface of short-stumpy forms [23]
6. Glycosomes in normal conditions but migrating to the endoplasmic reticulum in alkaline conditions [24].

Both groups 5 and 6 employed the same antibody, monoclonal anti-GPI-PLC immunoglobulin G (IgG), 2A6-6 [25] but observed different locations.

In the present study we determine the location of the GPI-PLC in a series of experiments that use a new polyclonal rabbit IgG anti-GPI-PLC antibody, specific for the GPI-PLC and extensively characterized. This robust antibody has been employed together with marker probes and antibodies directed against proteins of known location in conjunction with confocal microscopy, matrix assisted laser desorption ionization time of flight mass spectrometry (MALDI-TOF MS), non-penetrating surface radio-iodination and other techniques. The GPI-PLC was found to be located exclusively in the outer leaflet of the plasma membrane covering the flagellum and confined to a narrow linear array along the flagellar attachment zone.

## Results

### The GPI-PLC was detected in wild-type but not in GPI-PLC null mutant bloodstream form trypanosomes

**Figure 1:**
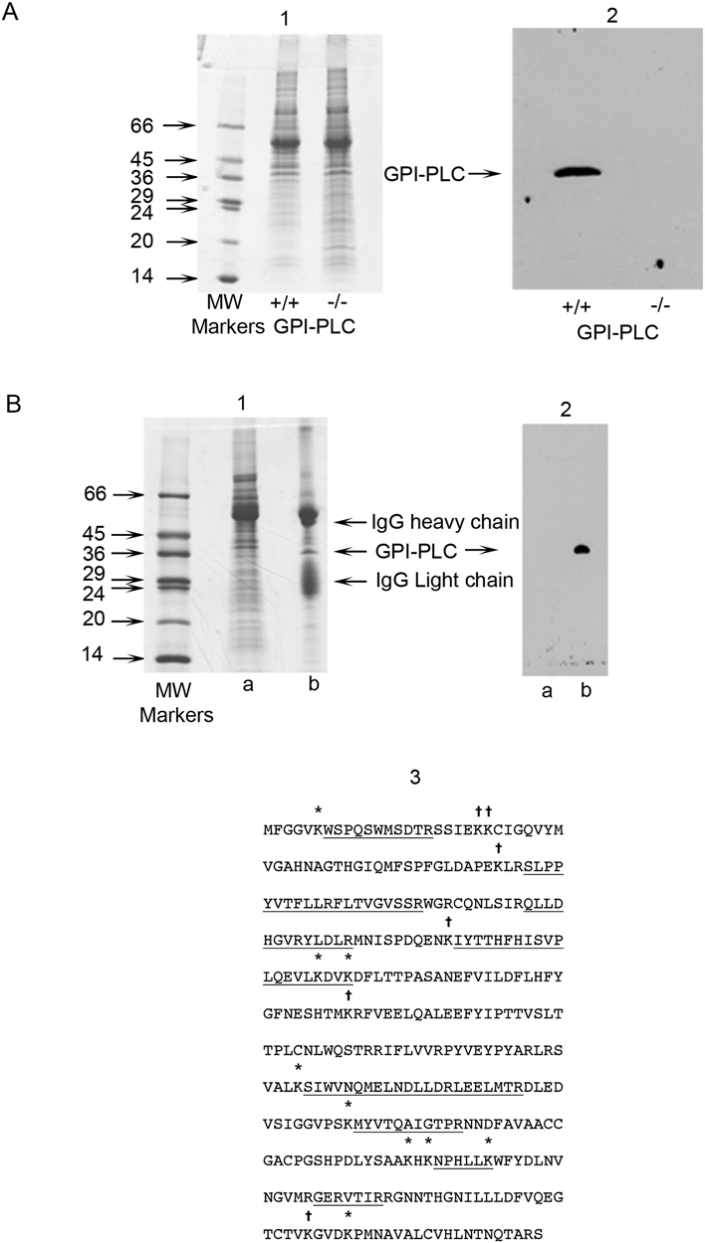
Specificity of the anti-GPI-PLC antibody for the GPI-PLC. Proteins in both wild type GPI-PLC^+/+^ and GPI-PLC^−/−^ null mutant trypanosomes were separated by SDS-PAGE on 15% (w/v) polyacrylamide gels and stained with Coomassie blue (panel A, image 1). The protein bands on replica gels were transferred to a PVDF membrane and probed with 3.9 µg of rabbit IgG anti-GPI-PLC primary antibody, followed by goat anti-rabbit IgG secondary antibody (panel A, image 2). The GPI-PLC was specifically immunoprecipitated from detergent lysates of whole bloodstream forms of *T. brucei* and was subjected to SDS-PAGE (panel B, image 1) and western blotting (panel B, image 2). In a parallel experiment the indicated 39 kDa protein band was cut from the gel, processed, digested with trypsin and then subjected to MALDI-TOF MS (panel B, image 3). In the protein sequence of the GPI-PLC the peptides detected are underlined. The symbol * above a lysine (k) represents either an unblocked lysine within the detected peptide or an unblocked lysine immediately preceeding the detected peptide, after which trypsin has successfully cleaved. The symbol † above a lysine (k) represents a position within the sequence that has not yet been determined by MALDI-TOF MS to be un-derivatized.

The specificity of the anti-GPI-PLC antibody was validated by Western blotting following SDS-PAGE. Coomassie blue staining revealed little if any detectable difference between wild-type and GPI-PLC null mutant cells (Figure 1, panel A, image 1, tracks +/+ and −/− respectively). However, the antibody detected a single protein band at approximately 39 kDa in wild-type cells, which was absent in the GPI-PLC null mutant cells (Figure 1, panel A, image 2, tracks +/+ and −/− respectively).

### The band immunoprecipitated with the anti-GPI-PLC antibody from wild-type trypanosomes was the GPI-PLC as shown by MALDI-TOF MS

The GPI-PLC in wild type bloodstream forms of *T. brucei* was immunoprecipitated using the anti-GPI-PLC antibody after extraction of cells with a mild neutral detergent mixture (see Methods). The immunoprecipitates were subjected to SDS-PAGE followed by Western blotting. The Coomassie stained gel revealed the presence of several bands. The two most prominent bands corresponded to the heavy chain (*M*_r_ 55000) and the light chain (*M*_r_ 23000) of IgG and a sharp band at 39000 corresponded to the *M*_r_ of the GPI-PLC (Figure 1, panel B, image 1, track b) which was absent from the immunosupernatant (Figure 1, panel B, image 1, track a). Several other weaker bands at higher *M*_r_ than the heavy chain of IgG were also present in the immunoprecipitate. When the bands from the immunoprecipitate in a replica gel were transferred to a polyvinylidene difluoride (PVDF) membrane and probed with the anti-GPI-PLC antibody, only the band corresponding to the GPI-PLC was detected (Figure 1, panel B, image 2, track b). This band was absent from the immunosupernatant (Figure 1, panel B, image 2, track a). The Coomassie blue stained band in a gel from a parallel experiment, corresponding to the 39 kDa protein band and recognized by the anti-GPI-PLC antibody in the Western blot, was cut from the gel, eluted, digested with trypsin and subjected to MALDI-TOF MS. The peptides detected were all derived from the GPI-PLC (Figure 1, panel B, image 3) with 31% sequence coverage, distributed uniformly throughout the protein.

In addition, MALDI-TOF MS also correctly identified the peptides in both portions of the intact GPI-PLC-MBP fusion protein and in the separated GPI-PLC and MBP after cleavage of the fusion protein (Text S1 and Figure S1).

### The GPI-PLC is exposed at the external surface of bloodstream forms of *T. brucei*

**Figure 2:**
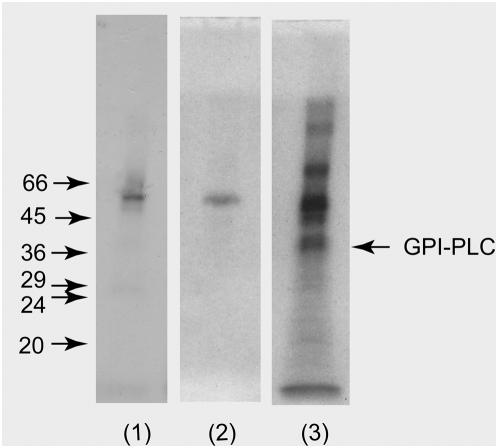
The GPI-PLC can be surface radio-iodinated in bloodstream form trypanosomes. Autoradiographs (15 day exposure) of the immunoprecipitates of the GPI-PLC in non-penetrating surface radio-iodinated GPI-PLC^+/+^ bloodstream forms (4.2×10^7^ cell equivalents per track) of *T.brucei* using either 95 µg of purified non-immune IgG with GPI-PLC^+/+^ (track 1), or using 77.8 µg of anti-GPI-PLC IgG to compare GPI-PLC^−/−^ (track 2) and GPI-PLC^+/+^ (track 3) bloodstream forms of *T. brucei*.

Immunoprecipitates were prepared from whole wild-type surface radio-iodinated Molteno Institute antigenic type (MITat) 1.2 cells and analysed by SDS-PAGE and autoradiography. No radio-labelled GPI-PLC was immunoprecipitated when preimmune IgGs were used (Figure 2, track 1). In this case only a very small amount of contaminating VSG was observed in the immunoprecipitate, which probably results from the large excess (approx. 200 fold) of VSG over that of the GPI-PLC in wild-type cells. No other proteins were detected when preimmune IgGs were used. In a separate but similar experiment the autoradiogram of the SDS-PAGE gel, containing the proteins in the anti-GPI-PLC immunoprecipitates of wild-type cells, displayed a strong band corresponding to the GPI-PLC as well as strong bands corresponding to several other proteins (Figure 2, track 3). However, when the same experiment was performed using GPI-PLC null-mutant cells, there was no band corresponding to the GPI-PLC in the autoradiogram (Figure 2, track 2). In addition, surface labelling of cells did not label the internal protein, tubulin (Text S1 and Figure S2). These experiments strongly suggest that the GPI-PLC is exposed to the external face of the plasma membrane.

### The GPI-PLC was detected in wild type bloodstream forms, both long-slender and short-stumpy types, but not in null mutant bloodstream forms or in procyclic forms by immunofluorescence

**Figure 3:**
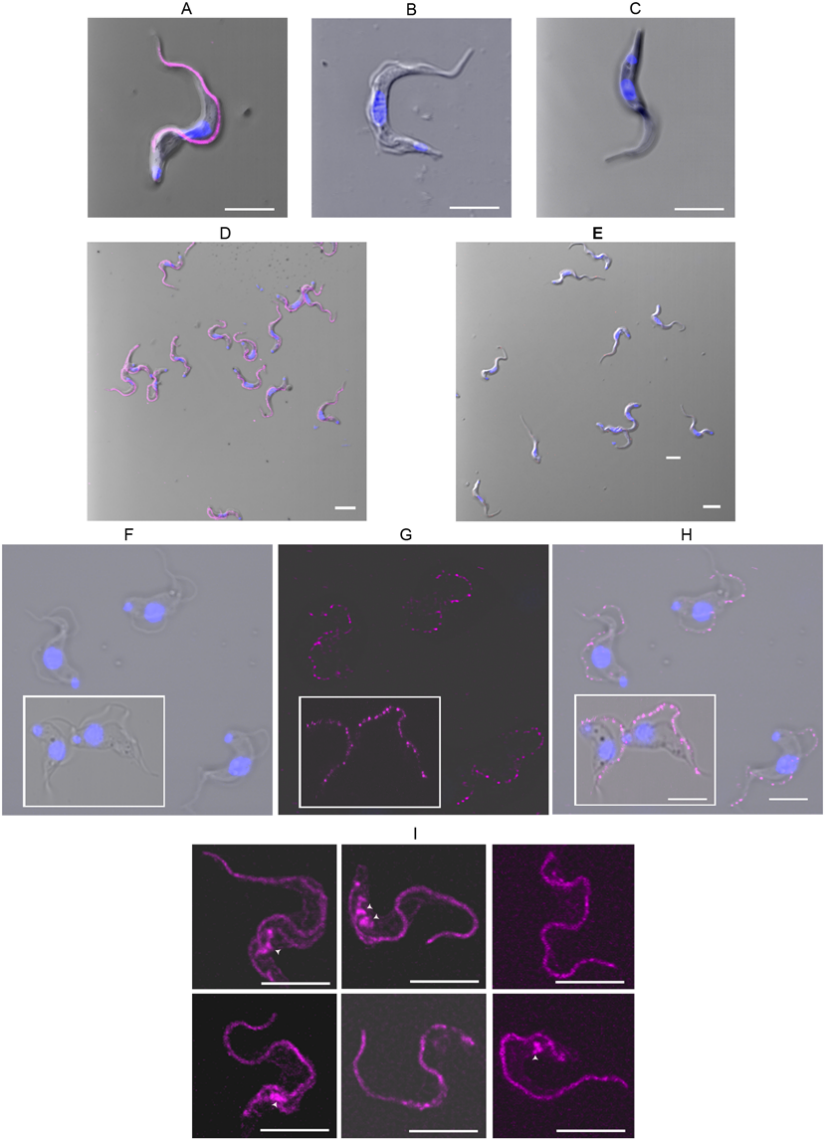
Confocal images of the GPI-PLC in GPI-PLC^+/+^, GPI-PLC^−/−^and procyclic form trypanosomes and the YFP-tagged GPI-PLC in recombinant bloodstream form cells. Each of the panels, A–E, contains a merged image of the nucleus and kinetoplast, together with the GPI-PLC probed with anti-GPI-PLC antibody (magenta) and the phase image; (panel A), wild-type GPI-PLC^+/+^ bloodstream form trypanosome; (panel B), a GPI-PLC^−/−^ null mutant bloodstream form trypanosome; (panel C), a wild-type procyclic form trypanosome; (panel D), a low power view of wild-type GPI-PLC^+/+^ bloodstream form trypanosomes and (panel E), a low power view of GPI-PLC^−/−^ null mutant bloodstream form trypanosomes. The phase image (panel F) of S-42 stumpy bloodstream forms shows the typical morphology of these forms in this strain, while the fluorescence image (panel G) shows the location of the GPI-PLC (magenta) and the merge of these two images shows their spatial relationship (panel H). The location of YFP-tagged GPI-PLC is shown in the composite image of six bloodstream forms in panel I and the arrowheads point to the endo-membrane-bound spaces containing a small amount of GPI-PLC. Bars, 5 µm.

The GPI-PLC was detected by immunofluorescence using the anti-GPI-PLC antibody in wild-type bloodstream forms (Figure 3, panel A) and was absent in GPI-PLC^−/−^ bloodstream forms (Figure 3, panel B) and in procyclic forms (Figure 3, panel C). The universal presence of the GPI-PLC was particularly striking in all wild type bloodstream form cells when viewed at low power magnification where many cells are found in the single field of view (Figure 3, panel D). Likewise the absence of the GPI-PLC in GPI-PLC null mutant bloodstream forms was obvious in all cells within the population in low magnification views (Figure 3, panel E). The distribution of the GPI-PLC in wild type cells was universally found in a single, patchy, linear array, along the line of flagellar attachment (Figure 3, panel A). It was never found in cytoplasmic vesicles, in the regions usually occupied by either the endoplasmic reticulum or the glycosomes. In addition it was never observed uniformly or even in a patchy distribution throughout the whole of the plasma membrane itself.

In natural infections, parasitaemias are generally pleomorphic with rapidly dividing slender forms that predominate in the ascending parasitaemia and short-stumpy forms in the descending parasitaemia [26],[27],[28]. The GPI-PLC was found to have the same pattern of fluorescence in both long-slender forms in the monomorphic strain, MITat 1.2 and short-stumpy forms in the very pleomorphic strain, S-42, but with the patchy nature of the linear array particularly striking in these short-stumpy forms (compare Figure 3, panels G and H with Figure 3, panel A).

### The YFP-tagged GPI-PLC location is identical to wild type GPI-PLC detected by immunofluorescence

In order to eliminate any possibility that the location of the GPI-PLC, determined by fluorescent antibody staining of cells, was due to an artefact, perhaps caused by reaction with a non-GPI-PLC epitope in bloodstream forms of *T. brucei* under the conditions employed for immunofluorescence, a gene encoding the GPI-PLC itself, tagged with yellow fluorecent protein (YFP), was introduced into cells and stably expressed. Its presence was detected by direct fluorescence of the YFP portion of the fusion protein itself. The location of the GPI-PLC was found by this alternate method to be identical to that determined by anti-GPI-PLC antibody staining. The GPI-PLC-YFP fusion protein was located mainly in the usual patchy linear array along the flagellum adjacent to the flagellar attachment zone (Figure 3I).

A much smaller fraction of the fluorescence was found within one or two small endo-membrane-bound cellular compartments (Figure 3I marked with arrowheads), anterior to the flagellar pocket but very close to the pellicular microtubules and the plasma membrane. The identity of this second compartment remains to be determined.

**Figure 4:**
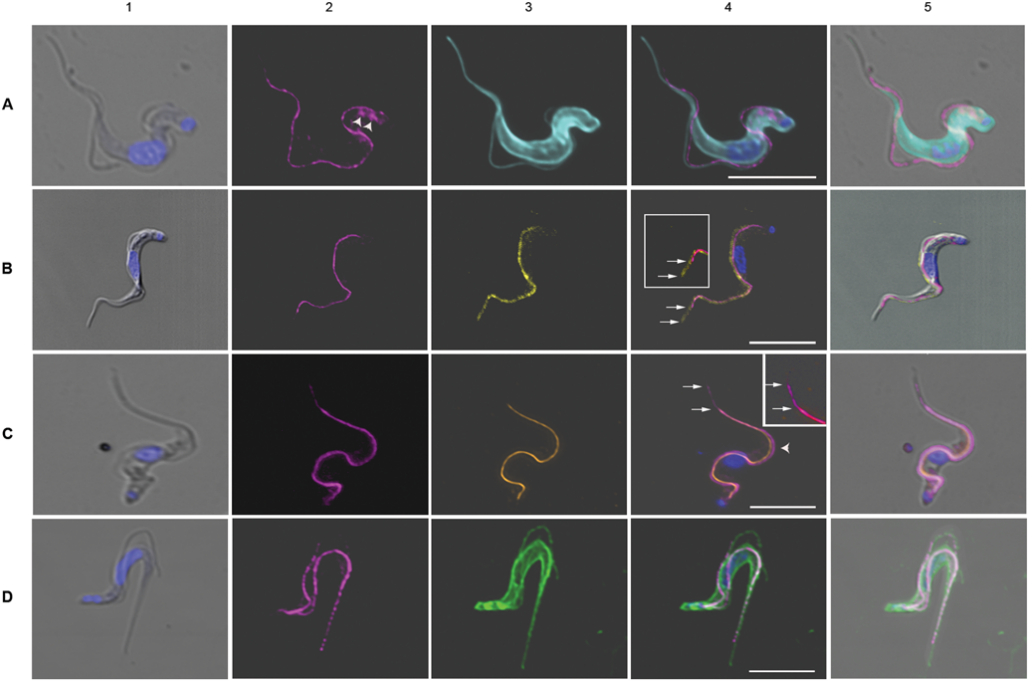
Location of the GPI-PLC compared to the location of a series of markers in *T. brucei*. Each panel shows the comparative locations of the GPI-PLC (magenta) and a marker; panel A, tubulin marker (cyan); panel B, paraflagellar rod marker (yellow); panel C, FAZ1 protein marker (orange); panel D, non-penetrating membrane protein reactive dye marker (green). (1), merge of the differential interference contrast image (DIC) and the Hoechst image of the nucleus and kinetoplast; (2), GPI-PLC; (3), marker; (4), merge of all images in the panel except the DIC image and (5) is the merge of all images. Arrowheads mark the location of a small amount of the GPI-PLC in a second endomembrane-bound compartment (Panel A-2). Upper large insets in panels B (4) and C (4) are the adjacent regions in panels B (4) and C (4) respectively with the saturation and brightness increased. Lower small insets in panels C (2), C (3) and C (4) are the adjacent regions in these panels with the contrast increased in the C (2) inset for clarity and contain small arrows marking the end of the structure containing both the GPI-PLC and the FAZ1 proteins. Upper arrows mark the end of the GPI-PLC linear array; lower arrows mark the end of the PFR in panel B (4) and the FAZ1 protein array in panel C (4). Arrowhead in panel C (4) indicates a region where separation of the FAZ1 protein and the GPI-PLC is particularly clearly demonstrated. Bars, 5 µm.

This second compartment has an interresting morphology. It is roughly flask-shaped with either a round or stocking-like body and a single neck that at one end is directed towards the plasma membrane. In some views the neck appears to be physically joined at its end to the plasma membrane. In a few cells this same location and morphology were also found by immunofluorescence (Figure 4A-2, see arrowheads). It may be significant that this structure and location were not found in all cells, whereas the linear array of the GPI-PLC on the flagellar membrane was a constant finding in all cells. For example, this second compartment may be present in only some stage(s) of the cell cycle. Furthermore, this observation together with its location and attachment to the plasma membrane may suggest that it is a structure involved in the assembly of the proteins destined to form the new flagellar attachment zone. In support of this possibility was the additional observation, using fluorescence-tagged antibodies, that this second structure also contained the FAZ1 protein, even though it was not entirely co-localized with the GPI-PLC (small arrows within the small lower insets in Figure 4 panels C-2, C-3 & C-4).

### Effect of temperature during fixation and the subsequent presence or absence of detergent on detection of the VSG, tubulin, ISG-70 and the GPI-PLC

**Figure 5:**
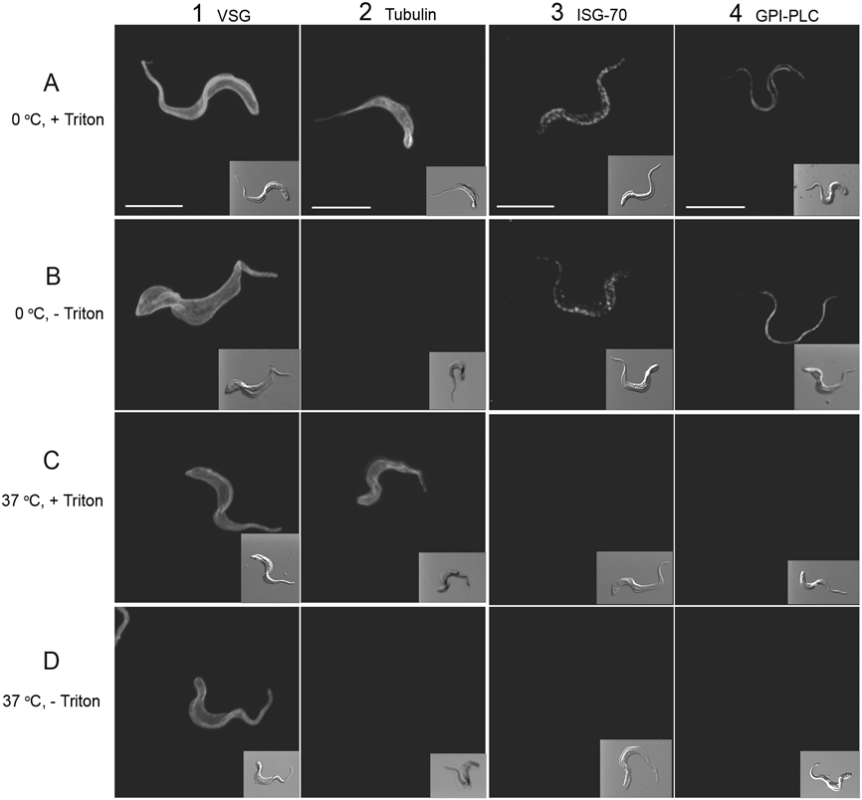
Effect of temperature and detergent on the detection of the VSG, tubulin, ISG-70 and GPI-PLC. Panel A shows the detection of VSG, tubulin, ISG-70 and GPI-PLC in wild type bloodstream form trypanosomes fixed at 0°C in the presence of triton X-100. Panel B shows the detection of VSG, tubulin, ISG-70 and the GPI-PLC in wild type bloodstream form trypanosomes fixed at 0°C in the absence of triton X-100. Panel C shows the detection of VSG, tubulin, ISG-70 and GPI-PLC in wild type bloodstream form trypanosomes fixed at 37°C in the presence of Tx-100. Panel D shows the detection of VSG, tubulin, ISG-70 and GPI-PLC in wild type bloodstream form trypanosomes fixed at 37°C in the absence of triton X-100. The inset in each panel is the Differential Interference contrast (DIC) image. Bars, 5 µm.

The VSG is the predominant surface protein in bloodstream form trypanosomes and forms the outermost surface of these cells, even shielding other exterior proteins. The VSG was evenly distributed over the entire surface of the trypanosome as reported previously by others [10],[29] and was visible when fixed, at either 0°C or 37°C in both the presence and absence of triton X-100 (Figure 5, panels A–D).

Tubulin is the major protein of microtubules in the trypanosomal cytoskeleton and is found as a pellicular array attached to the cytoplasmic face of the plasma membrane and as the flagellar axoneme enclosed by the flagellar plasma membrane. Therefore, mouse monoclonal IgG anti-α-tubulin (DM1A) antibody [30], followed by Alexa 488-conjugated anti-mouse IgG, identifies both the position of the inner-most surface of the plasma membrane of the cell body, as well as the axonemal portion of the flagellar compartment. However, this antibody combination detected tubulin in trypanosomes only when 0.1% triton X-100 was present during the blocking and antibody treatment steps regardless of whether trypanosomes were previously fixed at either 0°C or 37°C (Figure 5, panels A & C) and failed to detect tubulin in the absence of triton X-100 (Figure 5, panels B & D). The distribution of tubulin was found to produce a uniform fluorescence from FITC-labelled anti-tubulin along the inner surface of the cell body as well as along the central portion of the flagellum.

Invariant Surface Glycoprotein70 (ISG-70) is an externally disposed integral membrane glycoprotein, present only in bloodstream forms, and is found in a patchy distribution over the entire surface of the plasma membrane covering both the cell body and flagellum. The VSG from a number of different variants masks the presence of ISG-70 in live but not in fixed cells [31]. Probing fixed trypanosomes with purified polyclonal rabbit anti-ISG-70 primary antibody followed by Cy-3-labelled goat anti-rabbit IgG secondary antibody identifies the area of the outer leaflet of the plasma membrane shielded by the VSG. ISG-70 was found distributed over the entire surface in an irregular, patchy pattern when cells were fixed at 0°C, in both the presence and absence of triton X-100 (Figure 5, panels A & B). However, fluorescence was absent when trypanosomes were fixed at 37°C (Figure 5, panels C & D) in both the presence and absence of triton X-100.

The GPI-PLC was detected by rabbit polyclonal IgG anti-GPI-PLC, followed by Cy-3-conjugated goat anti-rabbit IgG, in cells fixed at 0°C (Figure 5, panels A and B), while fixation at 37°C resulted in no detectable fluorescence (Figure 5, panels C and D). Detection of the GPI-PLC was insensitive to the presence or absence of detergent. Clearly, the ability to detect the GPI-PLC follows the pattern displayed by ISG-70 with respect to temperature and detergent and does not resemble the pattern shown by either the VSG or tubulin, suggesting that the GPI-PLC is located in a restricted portion of the outer leaflet of the plasma membrane and at least partially shielded by the VSG in the same manner as ISG-70

### Comparative localization of the GPI-PLC and tubulin

In order to verify that the GPI-PLC was not localized to the inner face of the plasma membrane, its location with respect to tubulin was investigated. The distribution of tubulin was found to produce an intense fluorescence due to the large numbers and regular disposition of the subpellicular microtubules. The flagellum was seen as a fluorescent, wavy line along the side of the cell body, due to the microtubules of the flagellar axoneme (Figure 4, panel A, images 3 & 4). The GPI-PLC was found distributed between these two regions of microtubules lying closer to the flagellar axoneme than to the cell body (Figure 4, panel A, images 2 & 4) and at no point were the two fluorescent dyes co-localized. Furthermore, the GPI-PLC was distributed along the line of the flagellum, indicating that the GPI-PLC was not localized to the cell body but, rather, to the flagellar compartment. A small fraction of the GPI-PLC was also seen in a few cells within an endo-membrane-bound space close to the inner surface of the cell anterior to the flagellar pocket (see arrowheads in Figure 4, panel A, image 2).

### Comparative localization of the GPI-PLC and the paraflagellar rod (PFR)

The paraflagellar rod is a large structure found within the flagellum of bloodstream form trypanosomes, extending from the point where the flagellum exits the flagellar pocket, running alongside the axoneme of the flagellum to its distal tip, and is physically connected to the axoneme *via* fibres attaching the proximal domain to microtubule doublets 4 through 7 [32]. The crescent shape of the paraflagellar rod and its location between the axoneme and the flagellar attachment zone in trypanosomes prompted us to determine the localization of the GPI-PLC simultaneously with that of the paraflagellar rod.

The pattern of staining using anti-PFR-A confirmed past observations [32],[33]. The paraflagellar rod appeared as a slightly curved thick linear rod within the flagellar compartment adjacent to the axoneme, stretching from the lip of the flagellar pocket to the distal tip of the free flagellum (Figure 4, panel B, images 3 & 4). The GPI-PLC was detected in the same cells by treatment with rabbit IgG anti-GPI-PLC, followed by Cy-3-conjugated goat anti-rabbit IgG, and was found in a patchy, linear array, beginning at a point close to the lip of the flagellar pocket but slightly more distal than the proximal end of the paraflagellar rod, to an area just posterior to the tip of the free flagellum far beyond the end of the cell body but ending before the distal tip of the paraflagellar rod (Figure 4, panel B, images 2 & 4 and image 4 inset). The GPI-PLC was located adjacent to the paraflagellar rod on the side of the rod facing the cell body in the region where the flagellum is attached to the cell body (Figure 4, panel B, images 2 & 4). However, the localization of the GPI-PLC was clearly distinct from that of the paraflagellar rod. In fact the merged image (Figure 4, panel B image 4) shows that the two proteins can be resolved into two non-overlapping linear structures. This distribution is particularly striking when the flagellum appears to cross from one side of the cell body to the other side of the cell body; *e.g.* when the flagellum, running along the top of the cell body falls towards one or the other side of the cell body. In each case the paraflagellar rod lies furthest from the cell body and the GPI-PLC lies closest to the cell body (Figure 4, panel B, image 4). This result indicates that the GPI-PLC is not located in the same position as the paraflagellar rod but, rather, lies in the region between the paraflagellar rod and the cell body. Furthermore, the GPI-PLC (Figure 4, upper arrows in both panel B image 4 and in inset) does not extend along the free flagellum as far as the paraflagellar rod (Figure 4, lower arrows in both panel B, image 4 and in inset).

### Comparative localization of GPI-PLC and the flagellar attachment zone protein, FAZ1

The flagellar attachment zone (FAZ) is found in the cytoplasmic compartment of the cell body, lying adjacent to the paraflagellar rod, which is in the flagellar compartment, in bloodstream form trypanosomes [33] and is composed of a number of proteins. These published images prompted us to determine whether the GPI-PLC was located in the same compartment as the FAZ, because we found the GPI-PLC distributed immediately adjacent to the PFR on the cell body side of PFR (Figure 4, panel B, image 4), which is certainly close to the FAZ region. The fluorescence observed with mouse monoclonal IgG anti-FAZ1 protein antibodies (L3B2) followed by Alexa 488-conjugated goat anti-mouse IgG antibody (Figure 4 panel C images 3 and 4) was found in a region between the cell body and the flagellum (Figure 4, panel C, image 4 compare with Figure 4, panel C, image 5). The GPI-PLC was distributed in the usual patchy linear array along the outer side of the FAZ (Figure 4, panel C, image 4). The distribution of both the FAZ and the GPI-PLC begin close to the flagellar pocket, just anterior to the kinetoplast. However, the GPI-PLC (Figure 4, panel C, image 4, upper arrow and inset, upper arrow) extends beyond the distal tip of the FAZ (Figure 4, panel C, image 4, lower arrow and inset, lower arrow) into the region of the free flagellum. Even in the regions where both proteins are found, it is apparent that the GPI-PLC does not co-localize with the FAZ1 protein (Figure 4, panel C, image 4, arrowhead). The GPI-PLC was located immediately adjacent to the outer side of the FAZ on the same side as the flagellum. Combining the information from the results in Figure 4, panels B and C) one can conclude that the GPI-PLC lies between the flagellar attachment zone (FAZ) and the paraflagellar rod (PFR). Therefore, when the flagellum lies on its side, the FAZ is closest to the cell body and the GPI-PLC is furthest from the cell body (Figure 4, panel C, image 4). The only structures that lie between the FAZ and the paraflagellar rod are the plasma membrane of the cell body and the flagellar plasma membrane. However, the GPI-PLC only co-localizes with the proteins of the flagellar plasma membrane and not with the proteins of the plasma membrane surrounding the cell body (Figure 4, panel D, image 4). Consequently, we conclude that the GPI-PLC is located in the flagellar plasma membrane.

**Figure 6:**
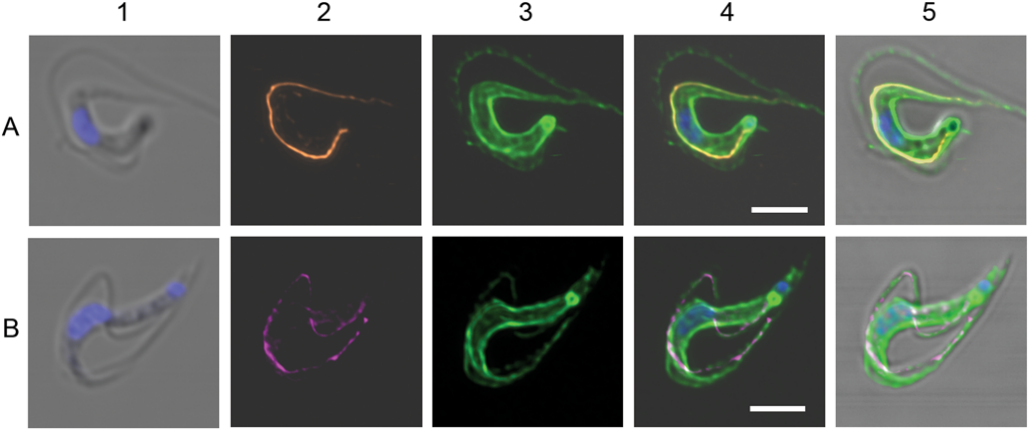
The location of GPI-PLC compared to a membrane marker and the FAZ1 protein in cells with a partially detached flagellum. Panel A shows the comparative location of the FAZ1 protein (orange) and a non-penetrating membrane protein reactive dye (green); (1), merge of the DIC image and image of the nucleus and kinetoplast; (2), FAZ1 protein; (3), membrane protein; (4), merge of all images in the panel except the DIC image and (5) is the merge of all images. Panel B shows the comparative location of the GPI-PLC (magenta) and a non-penetrating membrane protein reactive dye (green); (1), merge of DIC image and image of the nucleus and kinetoplast; (2), GPI-PLC; (3), membrane protein; (4), merge of all images in the panel except the DIC image and (5) is the merge of all images. The brightness of panel A image 4 and panel B, image 4 have been uniformly increased to the same extent for ease of viewing. Bars, 5 µm.

Figure 6, panels A & B are presented in order to provide additional evidence that firstly, the GPI-PLC and the FAZ1 protein are not in the same location and secondly, that the GPI-PLC is localized to the flagellar membrane of bloodstream form trypanosomes, while the FAZ1 protein remains with the cell body. The trypanosomes shown in Figure 6 have been selected because they are slightly damaged with their flagellum and surrounding membrane torn from their attachment to the plasma membrane of the cell body. These images distinguish clearly between the distribution of the GPI-PLC and the FAZ1 protein. The FAZ1 protein remains attached to the cell body (Figure 6, panel A, images 2, 4 and 5) while the GPI-PLC remains aligned along the flagellum (Figure 6, panel B, images 2, 4 and 5). Furthermore, the GPI-PLC was not distributed over all of the flagellar membrane. Rather, it occupied a position on the flagellar membrane immediately adjacent to the cell body (Figure 6, panel B, images 2, 4 and 5).

### Comparative localization of GPI-PLC and markers for the protein and lipid of the plasma membrane

The plasma membrane of bloodstream forms of *T. brucei* consists of three distinct but contiguous domains: the pellicular membrane, the flagellar membrane and the flagellar pocket membrane. Labeling trypanosomes with a Cy-3-dye conjugated to a maleimide reactive group, acts as a membrane protein marker and detects all three plasma membrane domains without labeling any internal membranes. It appears that the two negatively charged sulphonate groups and the positively charged quaternary nitrogen on the dye are sufficient to prevent entry of the dye into the cell, particularly in the presence of a negative plasma membrane potential, which excludes net negatively charged molecules lacking a specific energy-coupled transport system, *e.g.* the thiocyanate anion [34]. The cellular distribution of the GPI-PLC is shown in Figure 4, panel D, images 2, 4 and 5 in an actively dividing and, consequently, biflagellate cell. It is aligned along the length of both flagella. The Cy-3 dye has labeled both the plasma membrane of the cell body and the flagellar membrane (Figure 4, panel D, images 3, 4 and 5). When the images of the GPI-PLC and the plasma membrane fluorescence are merged the resulting image suggests that the GPI-PLC is located on the membrane of both flagella (Figure 4, panel D, image 4). The patchy linear distribution of the GPI-PLC localizes along the exterior portion of the membrane of the flagellum and is nowhere near the membrane of the cell body. Consequently, it appeared likely that the GPI-PLC was associated with the flagellar membrane. A similar result was obtained using the membrane surface lipid probe, fluorescein-derivatized dihexadecanoyl-glycerophosphoethanol amine [35] in conjunction with rabbit polyclonal IgG anti-GPI-PLC antibody and a Cy-3 conjugated goat anti-rabbit IgG secondary antibody (data not shown).

### Localization of the activated GPI-PLC after release of the VSG

**Figure 7:**
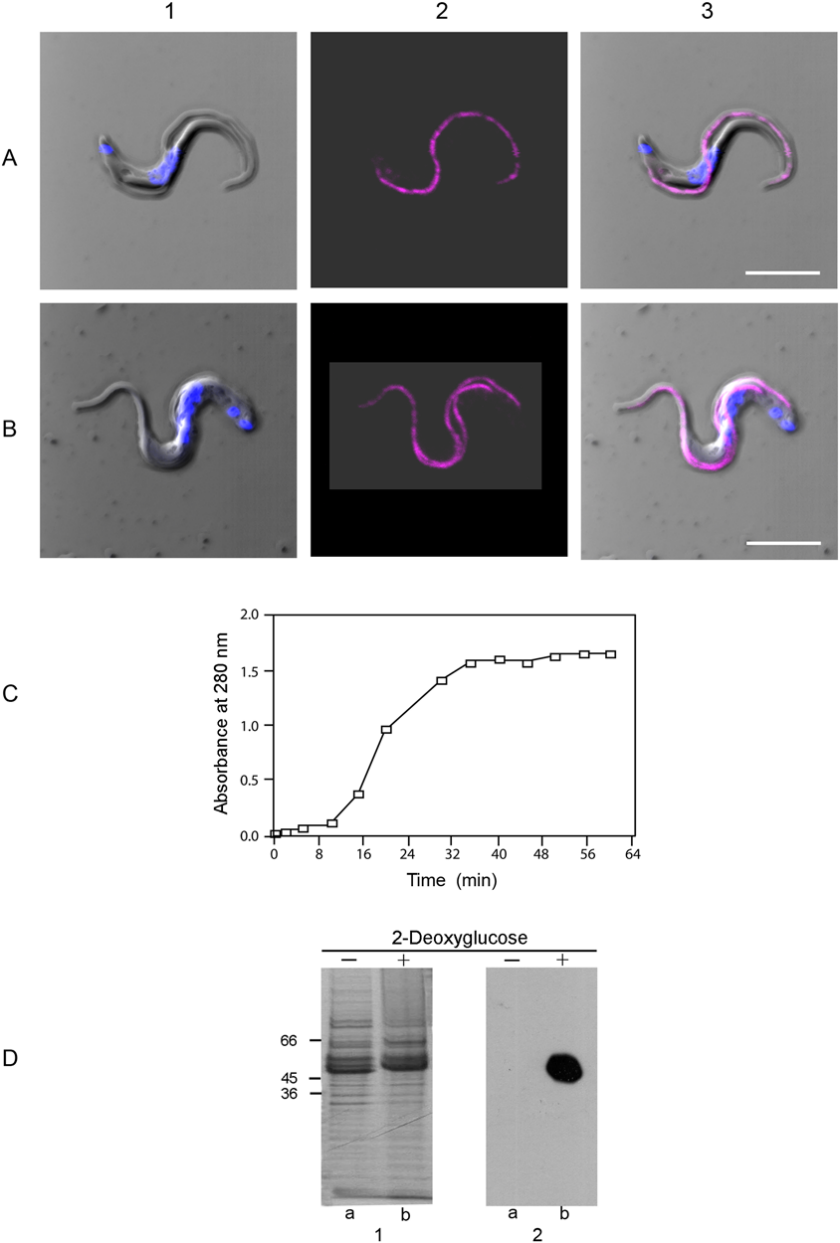
Location of the GPI-PLC in intact cells before and after release of the VSG. Panel A shows a cell in a fully energized trypanosome incubated for 45 min at 37°C in the presence of glucose (10 mM). Panel B, shows a cell incubated for 45 min at 37°C without glucose but with 2-deoxyglucose (10 mM) added. In each case panel (1) is a merge of the DIC (gray) and Hoechst stained nucleus and kinetoplast (blue) images, panel (2) is the GPI-PLC image (magenta) and panel (3) is a merge of all images. Panel C shows the time course of the release of the VSG from bloodstream form trypanosomes in the absence of glucose but in the presence of 2-deoxyglucose measured as absorbance of the supernatant of cells at 280 nm. Panel D, image 1 shows the Coomassie blue stained proteins after SDS-PAGE of bloodstream form trypanosomes (MITat 1.1) incubated for 60 min in the presence of glucose (track a) and in the absence of glucose but with 2-deoxyglucose added (track b). Panel D, image 2 shows an equivalent western blot. Bars, 5 µm.

An experiment was conducted to determine whether the activated GPI-PLC moved away from its previous position in the unactivated state. We have found that incubation of bloodstream form trypanosomes in the absence of glucose but in the presence of 2-deoxyglucose (10 mM) to de-energize cells and, further, in the presence of hyperosmotic phosphate - sucrose buffer to prevent cell rupture, activates the GPI-PLC and quantitatively releases the VSG over a 45 min time course. The distribution of the GPI-PLC in trypanosomes that have lost their VSG but are still intact was the same as it was in fully energized trypanosomes (Compare Figure 7, panel A, images 2 & 3 with panel B, images 2 & 3). These results suggest that the GPI-PLC does not move from its location in the flagellar membrane in order to cleave the GPI anchor of the VSG. In addition to the confocal microscopic analysis, protein release from the cell surface was measured (Figure 7, panel C) and the released protein analyzed by SDS-PAGE and western blotting (Figure 7, panel D). These results establish that the VSG was released from de-energized trypanosomes with a half time of 19 min (Figure 7, panel C), and converted from mVSG to sVSG as indicated by the paradoxical shift in apparent molecular weight [36] to an higher value (compare Figure 7, panel D, image 1, lanes a & b). Furthermore, conversion of the mVSG to the sVSG resulted in exposure of the CRD epitope on the sVSG, indicating that the release was due to cleavage of the VSG anchor by the GPI-PLC (compare Figure 7, panel D, image 2, lanes a & b). Consequently, release of the VSG occurred when the VSG diffused within the plane of the membrane to the position of the activated GPI-PLC and the activated GPI-PLC did not move to the position of each molecule of the VSG in turn.

## Discussion

Experimental results from this study do not support a localization of the GPI-PLC in the flagellar pocket, in the Golgi, on the cytoplasmic face of intracellular vesicles, in glycosomes or in the ER, as previously proposed [14],[24],[37]. This study does support the plasma membrane location reported originally by Turner [20]. However, our evidence also demonstrates that the GPI-PLC is mainly restricted to a small sub-domain of the plasma membrane covering the flagellum in both long-slender and short stumpy forms. A small part of the GPI-PLC in some long slender forms but not in short stumpy forms, is also found in an endo-membrane-bound space within the cell body, anterior to the flagellar pocket and both adjacent and connected to the flagellar attachment zone. It is not present in the plasma membrane of either the flagellar pocket or the cell body. Instead, it is disposed in a patchy, linear array along the flagellar membrane adjacent to or within the flagellar attachment zone, depending upon how this zone is defined. Furthermore, both the dependence of the detection of the GPI-PLC on the temperature of fixation and its independence of the presence of detergent as well as the results from non-penetrating surface labeling demonstrate that the enzyme is located in the outer leaflet of the plasma membrane, consistent with the report by Gruszynski et al. [23]. Importantly, the GPI-PLC remains in the same location, both before and after activation.

We have estimated the minimum amount of GPI-PLC that could be detected if present elsewhere in the cell. Clearly, detection depends upon the amount of material present and the surface area over which it is distributed. The relevant area is the projected area of the 3-dimensional site being considered, which is less than its true area because in the projected image, the top surface area is superimposed upon the lower surface area and the surface area of the sides are stacked edge-on. Consequently, the projected surface area is likely to be close to a quarter of the true surface area. However, the integrated pixel densities in the sum of a series of confocal slices are also coincident in the projected image. Therefore, the ratio of integrated pixel density to projected area is the relevant parameter for confocal microscopy. The area of the projected image of the GPI-PLC has been found to be 9.84±1.54 µm^2^ (n = 100) in re-assembled confocal images, while the area of the projected image of the plasma membrane has been found to be 37.2±4.7 µm^2^ (n = 100). The integrated pixel intensity of the GPI-PLC in the projected image under ideal conditions was 7,226,942.±189. This value dropped to 427,312.±22. in the image that was just visible when the gain was reduced from 640 v to 580 v in the final stage of the amplifier circuit. Consequently, if the GPI-PLC were spread over the entire plasma membrane, the minimum level of detection would be 22.4% of the amount of GPI-PLC actually detected. In contrast to this calculation, if the GPI-PLC were spread over the projected area of a different site, then the minimum level of detection would be a smaller % of that actually observed by a factor of
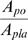
where *A_po_* is the projected area of the site containing the GPI-PLC and *A_pla_* is the projected area of the GPI-PLC itself. Therefore, if the GPI-PLC were truly in the glycosomes or in the flagellar pocket, our minimum level of detection would be 2.4% and 3.2% respectively of the GPI-PLC actually detected, where the projected area of all glycosomes in the cell was calculated to be 15.8 µm^2^ from the total glycosome area per unit cell volume [38] and the volume of a cell determined experimentally from [39], converted to projected total glycosome area by dividing by 4, which arises from the ratio of the area of a circle (πr^2^) divided by the area of a sphere (4πr^2^) when both have the same radius. The projected area of the flagellar pocket was calculated to be 1.67±0.508 µm^2^ (n = 100) from our own unpublished data. The way in which all of the preceeding calculations were performed is documented in the supplementary material (Text S1).

We have discovered that the temperature of fixation affects the detection of external, partially buried proteins. This observation has been used as a novel method to identify such proteins. Analysis of these temperature and detergent effects provided important evidence for the location of the GPI-PLC. For example, tubulin is located exclusively behind the permeability barrier of the plasma membrane, while the VSG is located on the exterior surface of the cell and the ISG-70 is located on the exterior surface but shielded by the VSG. Consequently, tubulin could be detected by immunofluoresence only when triton was present, while the VSG and ISG-70 were equally well detected in both the presence and absence of triton. The GPI-PLC was also detected equally well in both the presence and absence of triton, strongly suggesting that it, like the VSG and ISG-70, was located outside the cell.

In contrast to the differential effects of detergent, both tubulin and the VSG could be seen equally well when fixed at either 0°C or 37°C. However, detection of both ISG-70 and the GPI-PLC was strongly affected by the temperature during fixation. Both proteins were only detected well when cells were fixed at 0°C. Our interpretation of this finding depended upon the fact that the VSG partially masks access to underlying proteins on the cell exterior, which is a major function of the VSG. We reasoned that the VSG only blocked access of antibodies if it projected beyond underlying proteins and both diffused rapidly in the plane of the membrane and bent across shielded proteins at rates similar to those predicted for inter-bond flexing within proteins, which occur on an infra-red scale.

Composite results taken from structural studies of both the large N-terminal folding domain of MITat 1.2 VSG by x-ray crystallography [40] and its small C-terminal folding domain by NMR [41] have identified two unstructured, flexible regions within the peptide chain of the VSG, which may allow bending of the VSG on the cell surface. These regions comprise the hinge region (dodecapeptide: QKHKPAESQQQA) between the N- and C-terminal folding domains and the region at the extreme C-terminus of the small folding domain (hepadecapeptide: ETAKDGKTGNTNTTGSS) that links the VSG to its GPI-anchor. The peptide backbone of these flexible regions are shown as single lines within the space-filling cartoon of the VSG in Figure 1 - Scheme 1 panel A, illustrating the orientations that the VSG folding domains may adopt at 37°C, relative to each other and to the plane of the membrane, to block access of anti-GPI-PLC antibodies to the GPI-PLC.

**Figure 8:**
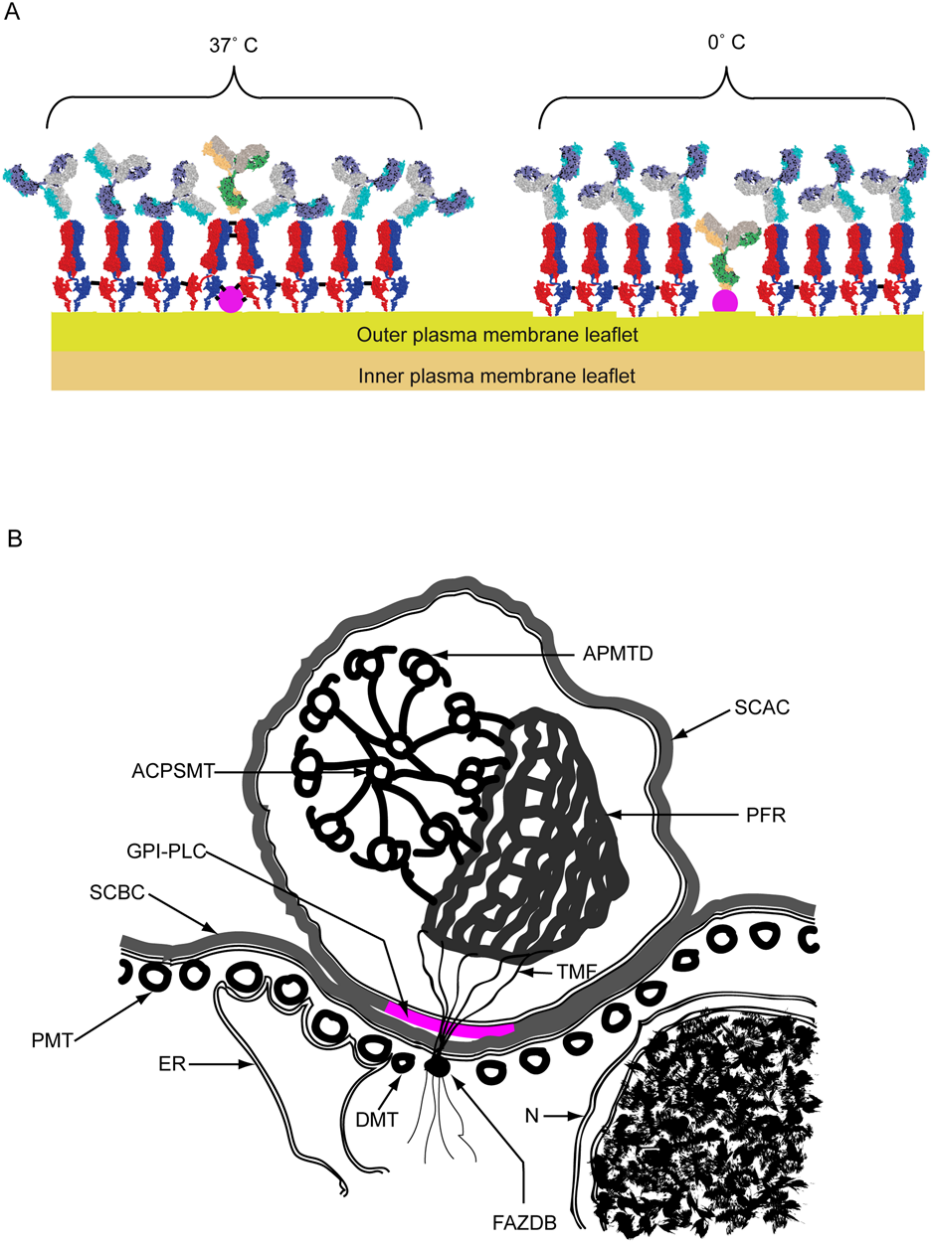
Scheme 1. Cartoons showing the arrangement of VSG dimers shielding the GPI-PLC at 37°C and allowing access to the GPI-PLC at 0°C as well as the cross-sectional relationships between the GPI-PLC and other surrounding structures. Panel A. Cartoon of the arrangement of VSG dimers (red & blue) on the outer leaflet of the plasma membrane of bloodstream form trypanosomes after mild cross-linking (black bars) of proteins by brief fixation (10 min) at either 37°C or 0°C. Subsequent access to the VSG is shown by the position of attached IgG anti-VSG (grey, mauve and turquoise) and access to a partially buried protein, such as the GPI-PLC (mauve), is shown by the position of attached or excluded IgG anti-GPI-PLC (yellow, green and grey). Panel B. Cartoon of a cross section through the cell body and flagellar compartment showing the relationships between the GPI-PLC and the flagellar attachment zone together with associated structures, including the ACPSMT, axonemal central pair single mictotubule; APMTD, axonemal peripheral microtubule doublet; DMT, diminished microtubule [29]; ER, loop of endoplasmic reticulum contacting the four constant microtubules within the flagellar attachment zone; FAZDB, flagellar attachment zone dense body; GPI-PLC (magenta), the glycosylphospatidyl-inositol phospholipase C; N, nucleus covered by inner and outer nuclear membranes; PMT, pellicular microtubule; PFR, paraflagellar rod; SCAC, surface coat covering the plasma membrane of the flagellar compartment; SCBC, surface coat covering the plasma membrane of the cell body compartment; TMF, one of the transmembrane fibrils connecting the paraflagellar rod to the flagellar attachment zone dense body.

Lowering the temperature from 37°C to 0°C slows the rates of both bond flexing and VSG diffusion. Consequently, the VSG spends short periods as partially coalesced aggregates within the plane of the membrane and access channels between the VSG condensates are transiently opened. Covalent fixation of cells at this lower temperature maintains the VSG in the partially aggregated condition and the channels of access permanently open, even if the temperature is subsequently raised, which allows the anti-GPI-PLC antibodies to reach their target (Figure 8 - Scheme 1 panel A). On the other hand, fixation of cells at 37°C allows adjacent molecules of VSG to be covalently linked across the upper surface of underlying proteins, creating an external lattice of cross-linked VSG dimers that serves as a permanent barrier against antibody access to underlying proteins (Figure 8 - Scheme 1 panel A). In some early experiments we explored the effect of other, intermediate temperatures of fixation between 0°C and 37°C on the ability to detect the GPI-PLC and found that it was a graded effect. Intermediate fixation temperatures gave image intensities that were detectable but less than that observed when fixed at 0°C, which would be expected for an effect that depended on VSG mobility. Further support for the idea that the temperature dependence of detection of the GPI-PLC is antibody access dependent comes from the observation that detection of the innate fluorescence of YFP-tagged GPI-PLC is not temperature dependent in this manner (data not shown).

This mechanism does not mean that antibodies against surface proteins, masked by the VSG, bind to their targets at 0°C in live cells. In the absence of fixative, the VSG on the surface of the cell, even at 0°C, is still in relatively rapid flux between the partially aggregated state and the dispersed state and can still bend across proteins underlying the VSG, preventing antibody binding.

The prediction from immunofluorescence observations that the GPI-PLC is exposed at the outer surface of the cell was confirmed by non-penetrating surface labeling with ^125^I, using the glucose oxidase/lactoperoxidase method. We have not found it possible to biotinylate the GPI-PLC under any conditions, even using the purified recombinant GPI-PLC, which differs from the results of Gruszynski *et al.* [23]. However, there was no difficulty in surface biotinylating other proteins.

The molecular weights of glucose oxidase and lactoperoxidase are just greater than that of the VSG monomer and 62% of the VSG monomer respectively, while the atomic weight of the iodide anion and its free radical are both less than 1/600 of the molecular weight of the VSG monomer and the molecular weight of H_2_O_2_ is less than 1/1700 of the molecular weight of the VSG monomer. Consequently, the mechanism preventing anti-GPI-PLC, glucose oxidase and lactoperoxidase from accessing the GPI-PLC, cannot prevent access of either the iodide anion and H_2_O_2_ or the iodine free radical, which reacts with tyrosines at the GPI-PLC surface [42],[43].

In cells where the flagellum has detached from the cell body, the GPI-PLC remains entirely with the flagellum. When this observation is combined with the information from co-localization and surface-labelling experiments, it can be safely concluded that the GPI-PLC is located in the outer leaflet of the flagellar plasma membrane, disposed in a patchy, linear array along the length of the flagellar attachment zone and extending into the free flagellum (Figure 8 - Scheme 1, panel B).

The present study resolves the topological issue that arose when the enzyme and its substrate were thought to be in separate compartments. Furthermore, placing the GPI-PLC on the outer leaflet of the plasma membrane makes the study of the regulation of the enzyme *in situ* of current interest. Consequently, the problem is not, “How does the GPI-PLC gain access to the VSG?” Rather, the question is, “What prevents the GPI-PLC from continuously releasing the VSG under basal conditions?” Clearly, the GPI-PLC must be tightly regulated and its restricted position suggests one possible mechanism. The GPI-PLC may have its active VSG binding site occluded by a partner protein under basal conditions but not under activated conditions. Certainly, the GPI-PLC must have at least one partner protein linked to the cytoskeleton, in order for it to be so strictly localized. Furthermore, activation of VSG release *via* the GPI-PLC in intact cells requires the presence of Ca^2+^ [8],[9], while the catalytic activity of the isolated enzyme itself is independent of Ca^2+^ [3] and does not possess a recognized Ca^2+^-binding motif. Consequently, either the partner protein or a separate regulatory protein may be a Ca^2+^-binding protein.

It is unlikely that the GPI-PLC is restricted in position and regulated by a caging mechanism without binding to a partner protein. Such a mechanism would require sufficient uncaging during activation of VSG release for the VSG to diffuse into the position of the GPI-PLC. Since the GPI-PLC is only 1/3 the size of a VSG dimer, activation of VSG release by uncaging would also allow diffusion of the GPI-PLC out of its restricted locale, which has not been observed. Consequently, it is difficult to reconcile the restricted location of the GPI-PLC without a direct attachment to some protein partner that interacts with the cytoskeleton.

The physiological role of the GPI-PLC in all cells is unclear. Deletion of the gene encoding the GPI-PLC did not exhibit any striking cellular phenotype in *T. brucei* [44]. However, there was a clear infection phenotype that was reversible by returning the gene. Recently this observation was extended by observing that the double knock-out of the genes encoding the GPI-PLC and a Zn^2+^-metalloprotease prevented differentiation from bloodstream forms into procyclic forms and also prevented cell proliferation, while deletion of either one of these two genes singly was without such an effect [19]. The possible role of the Zn^2+^-metalloprotease in physiological functions that also involve the GPI-PLC is of interest for two reasons.

First, differentiation of bloodstream forms into procyclic forms is accompanied by loss of the VSG and involves both the GPI-PLC and a protease acting at different times during differentiation. Both modes of release are independent and occur at the cell surface [23]. In addition, the VSG can be released by dividing bloodstream forms in culture, with a half-life of 32 hours [35],[45].

Second, treatment of BC_3_H1 myocytes with insulin also results in both cleavage of the GPI-anchor from GPI-linked alkaline phosphatase and proteolytic cleavage of the peptide link between its two folding domains [46]. Cell signaling *via* the small C-terminal peptide , following the sequential action of the protease and the GPI-PLC, has been suggested but has not been demonstrated [46],[47]. The phosphoinositolglycan has also been suggested to mediate cell signalling after its release from the GPI-anchor [48],[49] in mammalian cells.

The GPI-PLC has at least one particularly curious feature. It behaves as an integral membrane protein during purification [1],[3],[50], even though it has neither a leader sequence or any hydrophobic stretch spaning the membrane. Consequently, both its trafficking mechanism to the exterior and the way it is attached and remains restricted to a specific region must still be determined. The GPI-PLC lacks the SH4 domain that is both N- and S-acylated in the HASPB protein, which is trafficked to the plasma membrane in Leishmania by a novel non-classical mechanism [51],[52]. It has been suggested that the GPI-PLC is multiply S-acylated for attachment to the plasma membrane [53] but acylation does not explain how the GPI-PLC is restricted to a particular sub-domain of the plasma membrane nor how it is trafficked to this external location, unless multiple S-acylation, without the presence of an SH4 domain, also makes the GPI-PLC a substrate for an HASPB type of translocation.

## Materials and Methods

### Ethics statement

All animals were handled in strict accordance with good animal practice as defined by the relevant national and/or local animal welfare bodies, and all animal work was approved by the appropriate committee.

### Antibodies

Anti-GPI-PLC IgG and anti-ISG-70 were prepared by the popliteal lymph node route in rabbits [54] using either recombinant GPI-PLC [55] or purified ISG-70 [31]. After 3 subcutaneous boosts at 2-week intervals crude anti-sera was prepared, divided into 3 ml samples, frozen and stored at −20°C. Rabbit IgG anti-MITat 1.2 VSG was prepared by the procedure of O'Beirne & Voorheis [56]. All crude anti-sera were purified by elution from a protein A-Sepharose column [57]. Rabbit anti-CRD IgG was prepared by immunization with soluble ILTat 1.21 VSG. Cross-reacting antibodies were then eluted after absorption to MITat 1.6 VSG coupled to cyanogens bromide (CNBr)-activated Sepharose [58]. Anti-PFR-A and anti-FAZ1 antibody (L3B2) were kind gifts from Prof. Keith Gull (Sir William Dunn School of Pathology, University of Oxford, England). Anti-α-tubulin IgG (Clone DM1A) was obtained from Sigma. Alexa 488-labeled secondary antibodies were from Invitrogen (Molecular Probes). Cy-3-labeled secondary antibodies were from Jackson Immunoresearch.

For immunoprecipitation, protein-A purified rabbit anti-GPI-PLC IgG (156 µg in 0.02 ml) was bound to Protein A-Sepharose beads (0.02 ml; 50% v/v; in PBS buffer, pH 7.5, 136 mM NaCl, 3 mM KCl, 16 mM Na_2_HPO_4_, 3 mM KH_2_PO_4_) by incubation with rotational mixing overnight at 4°C. The protein A-Sepharose beads with attached antibody were washed twice with PBS buffer, pH 7.5, containing 15 mM sodium azide and once with lysis buffer (50 mM Tris, pH 7.4, 150 mM NaCl, 0.1% NP-40, 0.5% CHAPS, containing 0.1 mM tosyl-lysine chloromethyl-ketone and 0.3 mM phenylmethylsulphonylfluoride) and finally resuspended to 0.04 ml (∼50% v/v slurry of beads) in lysis buffer.

### Generation of the GPI-PLC null mutant

Both alleles of the GPI-PLC gene in the Molteno Institute trypanosomal antigenic repertoire (MITar) serodeme were deleted using two constructs in a manner similar to that used previously for the AnTar serodeme: first, with pLN [44] and second, with pLH, which was generated by replacing the GPI-PLC gene in p1172kxL with a hygromycin cassette as a KpnI-Xbal fragment [44]. Bloodstream forms of the MITar serodeme, derived originally from Lister 427 [59] were grown in culture at 37°C in HMI-9 medium [60], containing 10% (v/v) heat inactivated foetal bovine serum. These cells were electroporated twice in the presence of BglI-linearized DNA. First, the cells were brought to hygromycin resistance using 5 µg pLH in a standard electroporation [44]. Second, after selection using hygromycin (5 µg/ml), the uncloned population was electroporated in the presence of 5 µg pLN. A doubly resistant population was selected in the presence of both hygromycin and G418 (2.5 µg/ml) and then cloned by limiting dilution. The cloned population stably expressed MITat 1.2 VSG and was negative for expression of GPI-PLC by Western blotting.

### GPI-PLC-YFP fusion protein

The GPI-PLC was expressed as a fusion protein in frame with YFP at the C-terminus by modifying the endogenous locus in the GPI-PLC+/− heterozygote using the method described in [61]. After transfection, cell lines were selected for blasticidin resistance and the resulting fluorescent clones analysed. All of the several clones isolated gave similar patterns of fluorescence. For microscopy, ∼1×10^6^ log phase bloodstream form trypanosomes were recovered by centrifugation and resuspended in 10 µl of 0.02% formaldehyde in HMI-9 without serum at 25°C.

### Recombinant GPI-PLC-*malE* fusion protein

The gene encoding the GPI-PLC was amplified by PCR, using the ‘Expand’ high fidelity DNA polymerase system, from genomic DNA derived from *T. brucei* MITat 1.1 using a forward primer (5′-AGG GAT CCT TTG GTG TAA AGT GGT CAC CGC AG-3′) containing a BamH1 restriction site (underlined) and a reverse primer (5′-AGC AAC TCG AGT TAT GAC CTT GCG GTT TGG TTG GT-3′) containing an XHO1 restriction site (underlined) and then gel purified. This purified DNA was ligated into the *lacZa* gene within the cloning site of the similarly restricted, gel purified pMBP-parallel2 vector downstream of the *malE* gene, using T4 DNA ligase. The vector itself was the kind gift of Dr. Amir Khan, School of Biochemistry and Immunology, Trinity College Dublin and had been constructed by replacing the protease site in the linker fragment of a pMAL-c2 vector (New England Biolabs) with a tobacco etch virus (TEV) cleavage site. The sequence of the purified cloned product was found to be correct by DNA sequencing. The pMBP vector containing the GPI-PLC was transformed into *E. coli* BL21(DE3), expressed following addition of IPTG, and the fusion protein purified first by binding to an amylose resin column, eluted in the presence of maltose (10 mM), and then by chromatography on a Sephacryl S-200 column.

### Growth and isolation of trypanosomes

Growth, isolation and counting of bloodstream forms of *T. brucei* (MITat 1.1) were performed as described previously [62].

### Surface labeling

Bloodstream forms of *T. brucei* (10^8^ cells) were surface radioiodinated (1 mCi carrier-free sodium ^125^Iodide) using glucose oxidase (0.66 units) and lactoperoxidase (0.165 units) for 10 min at 25°C as described previously [31] and surface biotinylated (5×10^7^ cells) using sulfo-*N*-hydroxy-succinamide long chain spacer arm (NHS-LC)-biotin (0.3 mg) in a final volume of 1.1 ml for 20 min at 0–4°C essentially as described by Gruszynski *et al.* [23].

### Immunoprecipitation of GPI-PLC from bloodstream forms of *T. brucei*

Trypanosomes (5×10^8^ cells/ml) were washed twice in TSB buffer (44 mM NaCl, 5 mM KCl, 3 mM NaH_2_PO_4_, 118 mM sucrose, 10 mM glucose and 0.2 mM adenosine, pH 8). The cell pellet was resuspended in lysis buffer (50 mM Tris, pH 7.4, 150 mM NaCl, 0.1% NP-40, 0.5% CHAPS, containing 0.1 mM tosyl-lysine chloromethyl-ketone and 0.3 mM phenylmethylsulphonylfluoride) and incubated for 30 min on ice. Cell lysates were centrifuged at 15,000 ***g*** for 15 min and the supernatant collected. GPI-PLC was immunoprecipitated with anti-GPI-PLC IgG bound to protein A-Sepharose beads. The immune complexes were removed from the beads by boiling for 2 min in SDS and their constituent proteins separated by SDS-PAGE and stained with Coomassie blue. The protein band corresponding to the GPI-PLC (∼39 kDa) was cut out, the protein passively eluted and concentrated by a minor modification of the method of Wessel and Flugge [63], digested with trypsin and analyzed by MALDI-TOF mass spectrometry. All MS data were acquired using a Voyager-DE PRO MALDI-TOF mass spectrometer equipped with a nitrogen laser.

### Release of VSG from the surface of bloodstream form trypanosomes

Trypanosomes (5×10^7^/ml) were incubated (15 min) in PBS buffer (NaCl, 136 mM; KCl, 3 mM; Na_2_HPO_4_, 16 mM; sucrose, 40 mM; glucose, 10 mM; adenosine, 0.1 mM; pH 7.5) at 37°C in a final volume of 10 ml with constant gentle stirring to allow any adsorbed serum proteins to be removed by endocytosis. The incubation was terminated by adding an excess volume (5×) of ice-cold PBS buffer and centrifuging (9,000 ***g***×10 seconds at 4°C) in a minifuge. The pellets were resuspended in ice-cold iso-osmotic phosphate buffer (Na_2_HPO_4_, 20 mM; sucrose, 360 mM; pH 7.5) containing protease inhibitors (0.3 mM phenylmethylsulphonylfluoride, 0.1 mM tosyl-lysine chloromethyl-ketone, 0.1% (w/v) leupeptin & 0.2% (w/v) pepstatin), at a final concentration of 5×10^8^ cells/ml. The cells (5×10^8^ cells) were then diluted into a second phosphate buffer without glucose (Na_2_HPO_4_, 20 mM; sucrose, 360 mM; pH 8.0) containing the same protease inhibitors and 2-deoxy-**D**-glucose (10 mM) at 37°C to give a final cell concentration of 5×10^7^/ml and incubated at 37°C for 45 min. The incubation was terminated by a 10-fold dilution into ice-cold phosphate buffer (Na_2_HPO_4_, 20 mM; sucrose, 360 mM; pH 7.5) and the cells in samples of the suspension collected by centrifugation. The supernatants containing the released VSG were concentrated by Vivaspin membrane ultra filters (5 kDa cut-off). Both the cell pellets and the supernatants were subjected to SDS-PAGE and subsequent western blotting. For confocal microscopy, cells were fixed for 10 min at 0°C by mixing with equal volumes of fixative in the same buffer solution at a final concentration of paraformaldehyde of 3% (w/v).

### Immunofluorescence

Bloodstream form trypanosomes (5×10^7^ viable cells/ml) were resuspended in PBS buffer and incubated at 37°C for 20 min with constant gentle stirring and then a first set of samples were removed and fixed for 10 min at 37°C with 3% (w/v) paraformaldehyde final concentration. A second set of samples were removed and cold quenched immediately by adding an excess (5×) of ice-cold PBS buffer, centrifuged (11,000 ***g***, 2 min, 4°C) and the cells resuspended in PBS buffer and subsequently fixed for 10 min at 0°C with 3% (w/v) paraformaldehyde. Procyclic form trypanosomes were fixed for 30 min at 0°C. Following fixation, cells were washed twice in PBS buffer containing sodium azide (15 mM) before allowing the cells to settle on poly-**L**-lysine coated coverslips surrounded by thin nail varnish dams. Adherent cells were blocked with PBS containing 5% (w/v) BSA and 0.1 M methylamine, +/−0.1% (w/v) triton x-100 for 1 hr at room temperature. Cover slips with adherent blocked cells were incubated with the primary antibody in PBS containing 5% (w/v) BSA, +/−0.1% triton X-100 overnight at room temperature, followed by incubation with the secondary antibody in 5% BSA for 1 hr at room temperature. After two washes with PBS, the coverslips were mounted onto slides and sealed. The mounting media was 50% glycerol in PBS containing 4% (w/v) propylgallate anti-quenching agent. The DNA stain, Hoechst 33342, was added to the mounting media at 0.1 µg/ml to visualize the nucleus and kinetoplast.

For visualization of the paraflagellar rod and the FAZ, cells were settled on poly-**L**-lysine coverslips and fixed with cold methanol (−20°C) for 10 min, rehydrated with PBS and blocked with 5% (w/v) bovine serum albumin in PBS for 1 hr at room temperature as described by Sherwin *et al.* [64].

For labelling the protein in the outer leaflet of the plasma membrane of bloodstream forms of *T. brucei*, cells (5×10^7^ cells/ml) were incubated for 5 min at 30°C with a Cy-3 protein-reactive cyanine fluor (Amersham Cat. No. PA23001) that contains two negatively charged sulphonate groups and one positively charged quaternary nitrogen to prevent cell entry. Cells were then cold-quenched with 5 volumes of ice-cold PBS buffer and fixed at 0°C for 10 min with 3% (w/v) paraformaldehyde.

Primary antibodies and dilutions used were rabbit anti-GPI-PLC at 1∶1000, rabbit anti-VSG at 1∶1000, rabbit anti-ISG-70 at 1∶500, mouse anti-tubulin at 1∶350, mouse anti-PFR-A at 1∶50 and mouse anti-FAZ1 (L3B2) at 1∶5. Alexa 488 and Cy-3 -labeled secondaries were used at 1∶1000.

The GPI-PLC was probed with purified polyclonal rabbit anti-GPI-PLC IgG primary antibody. Tubulin was probed with mouse monoclonal anti-α-tubulin primary antibody (clone DMIA). The paraflagellar rod was probed with mouse monoclonal anti-paraflagellar rod-A IgG primary antibody [33]. The FAZ1 protein was probed with mouse monoclonal anti-FAZ1 IgG primary L3B2 antibody [33]. MITat 1.2 VSG was probed with purified rabbit polyclonal anti-MITat 1.2 VSG primary antibody. ISG-70 was probed with purified rabbit polyclonal anti-ISG-70 IgG antibody. All mouse monoclonal IgG primary antibodies were visualized using Alexa-488-conjugated goat anti-mouse IgG secondary antibody. All purified rabbit polyclonal IgG primary antibodies were visualized with Cy-3-conjugated goat anti-rabbit IgG secondary antibody.

Microscopy was performed on an Olympus FV1000 laser scanning Confocal Microscope, using an UPlanSAPO 60×/1.35 NA oil objective. Brightness and contrast adjustments were made to the whole digital image files and cropping images to size were both performed using Photoshop 9 (Adobe). Digital blind deconvolution of multichannel 3D microscopic images was performed with Bitplane Autodeblur software. Cells were digitally sectioned by confocal laser scanning fluorescence microscopy at 0.3 µm per slice and the composite Z-stack of all sections is shown in each figure.

## Supporting Information

Figure S1 Specificity of the anti-GPI-PLC antibody for the GPI-PLC within the recombinant MBP-GPI-PLC fusion protein. The purified maltose binding protein-GPI-PLC (MBP-GPI-PLC) fusion protein and the TEV cleaved purified MBP-GPI-PLC fusion protein was subjected to SDS-PAGE (panel A, image 1) and western blotting (panel A, image 2). In a second experiment, the band corresponding to the purified fusion protein and that corresponding to the GPI-PLC, derived by TEV cleavage of the recombinant immunoprecipitated fusion protein, were cut from the gel, eluted, trypsin cleaved and subjected to MALDI-TOF MS. Peptides corresponding to the GPI-PLC derived from the fusion protein are shown (single underlined) in panel B and gave 50% sequence coverage of the GPI-PLC. Peptides corresponding to the maltose binding protein from the fusion protein are also shown (double underlined) in panel B and gave 51% sequence coverage of the maltose binding protein. Peptides corresponding to the GPI-PLC, derived by TEV cleavage of the purified fusion protein, are shown in panel (C) and gave overall sequence coverage of 23%.
(1.01 MB TIF)

Figure S2 Immunoprecipitation of cytoplasmic tubulin in detergent lysates of surface labelled cells. Pleomorphic populations of ILTat 1.1 (5×10^7^ cells/ml) were surface biotinylated and the excess biotinylation reagent inactivated with 5 mM glycine, cells detergent-lysed, lysates centrifuged and the clear supernatants subjected to immunoprecipitation as described in Methods. Soluble tubulin was removed with anti-tubulin IgG bound to protein A-Sepharose beads and, following a wash, the immune complexes were removed by boiling for 2 min in SDS sample buffer and their constituent proteins separated by SDS-PAGE followed by Western blotting using (Panel A) anti-tubulin primary antibody followed by horseradish-conjugated secondary antibody or (Panel B) horseradish-conjugated streptavidin. In each case lane 1 contains the supernatant of a cell lysate (2×10^7^ cell equivalents); lane 2 contains the immunoprecipitated protein (2×10^8^ cell equivalents); lane 3 contains the supernatant of the immunoprecipitation reaction (2×10^8^ cell equivalents).
(0.17 MB TIF)

Text S1 Supporting information results and figure legends.
(0.04 MB DOC)
