# Supplementary figures and images for: The Glycosylphosphatidylinositol-PLC in Trypanosoma brucei Forms a Linear Array on the Exterior of the Flagellar Membrane Before and After Activation
Source: PLoS Pathog. 2009 Jun 5;5(6):e1000468. doi: 10.1371/journal.ppat.1000468 (PMC2685982; doi:10.1371/journal.ppat.1000468)

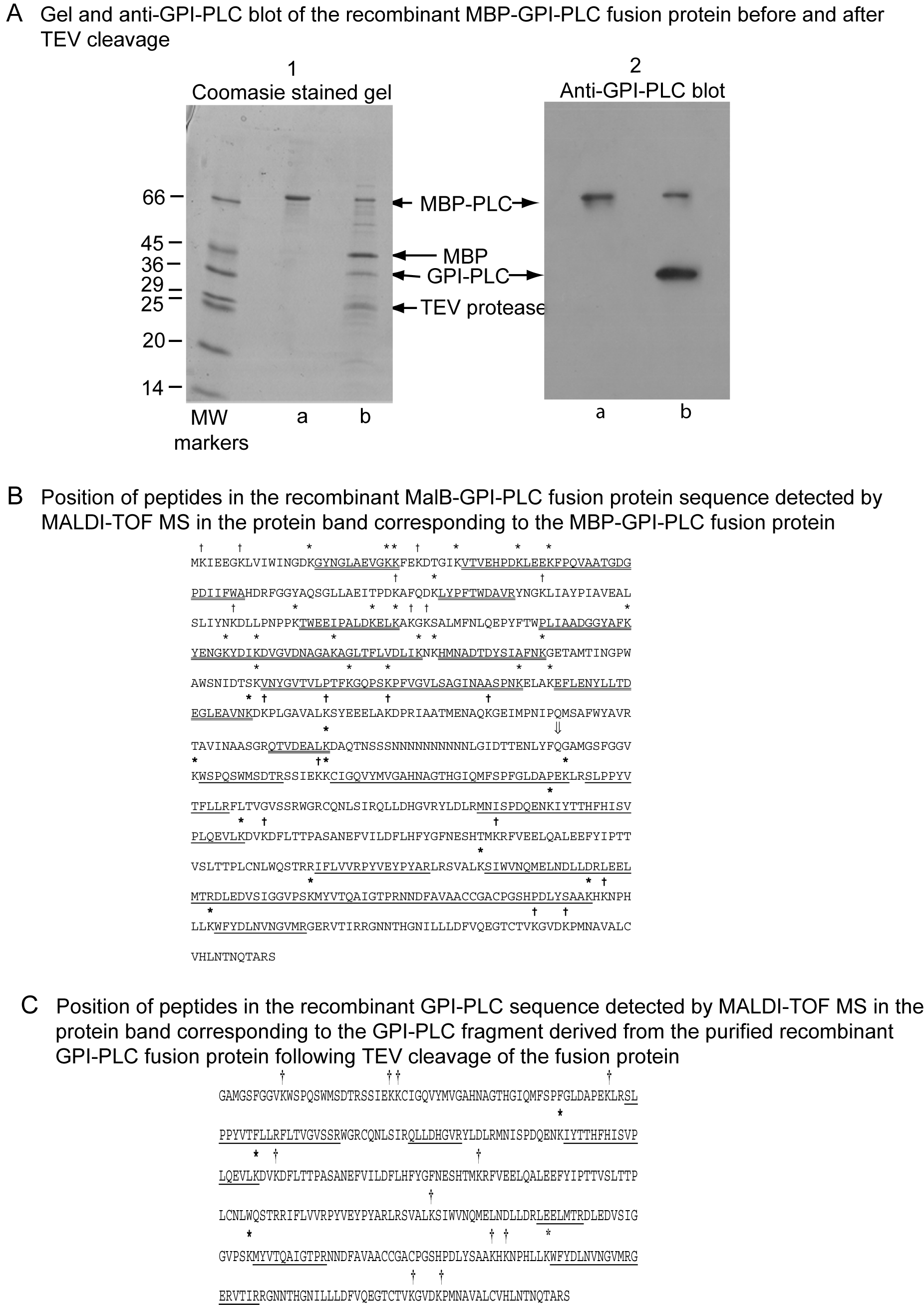

Supplement: Figure S1 — Specificity of the anti-GPI-PLC antibody for the GPI-PLC within the recombinant MBP-GPI-PLC fusion protein. The purified maltose binding protein-GPI-PLC (MBP-GPI-PLC) fusion protein and the TEV cleaved purified MBP-GPI-PLC fusion protein was subjected to SDS-PAGE (panel A, image 1) and western blotting (panel A, image 2). In a second experiment, the band corresponding to the purified fusion protein and that corresponding to the GPI-PLC, derived by TEV cleavage of the recombinant immunoprecipitated fusion protein, were cut from the gel, eluted, trypsin cleaved and subjected to MALDI-TOF MS. Peptides corresponding to the GPI-PLC derived from the fusion protein are shown (single underlined) in panel B and gave 50% sequence coverage of the GPI-PLC. Peptides corresponding to the maltose binding protein from the fusion protein are also shown (double underlined) in panel B and gave 51% sequence coverage of the maltose binding protein. Peptides corresponding to the GPI-PLC, derived by TEV cleavage of the purified fusion protein, are shown in panel (C) and gave overall sequence coverage of 23%. (1.01 MB TIF) [file ppat.1000468.s001.tif]

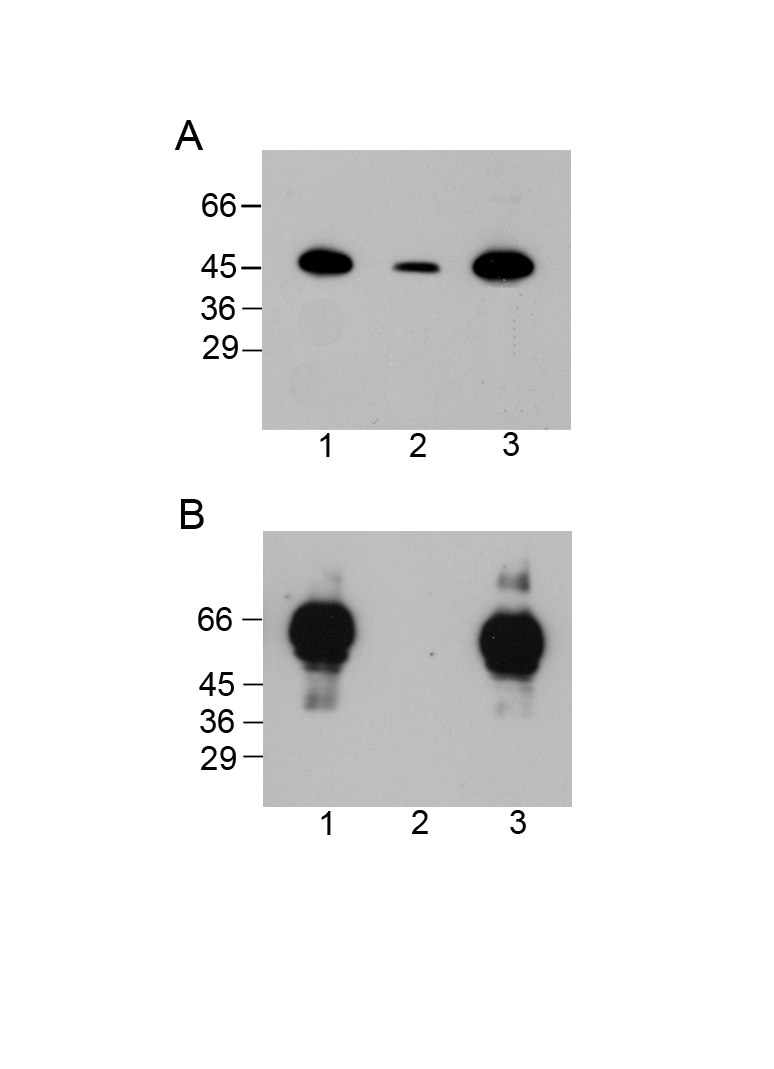

Supplement: Figure S2 — Immunoprecipitation of cytoplasmic tubulin in detergent lysates of surface labelled cells. Pleomorphic populations of ILTat 1.1 (5×10^7^ cells/ml) were surface biotinylated and the excess biotinylation reagent inactivated with 5 mM glycine, cells detergent-lysed, lysates centrifuged and the clear supernatants subjected to immunoprecipitation as described in Methods. Soluble tubulin was removed with anti-tubulin IgG bound to protein A-Sepharose beads and, following a wash, the immune complexes were removed by boiling for 2 min in SDS sample buffer and their constituent proteins separated by SDS-PAGE followed by Western blotting using (Panel A) anti-tubulin primary antibody followed by horseradish-conjugated secondary antibody or (Panel B) horseradish-conjugated streptavidin. In each case lane 1 contains the supernatant of a cell lysate (2×10^7^ cell equivalents); lane 2 contains the immunoprecipitated protein (2×10^8^ cell equivalents); lane 3 contains the supernatant of the immunoprecipitation reaction (2×10^8^ cell equivalents). (0.17 MB TIF) [file ppat.1000468.s002.tif]
