# Supplementary material for: The Glycosylphosphatidylinositol-PLC in Trypanosoma brucei Forms a Linear Array on the Exterior of the Flagellar Membrane Before and After Activation
Source: PLoS Pathog. 2009 Jun 5;5(6):e1000468. doi: 10.1371/journal.ppat.1000468 (PMC2685982; doi:10.1371/journal.ppat.1000468)
Supplement: Text S1 — Supporting information results and figure legends. (0.04 MB DOC) [file ppat.1000468.s003.doc]

**Supporting Information Results**

**Identification by MALDI-TOF MS of the peptides in the intact GPI-PLC-MBP fusion protein and in the separated GPI-PLC and MBP following cleavage of the fusion protein.**

The GPI-PLC was also expressed as a fusion protein with the maltose binding protein (MBP). The purpose of this experiment was to justify using the fusion protein as a source of GPI-PLC as an antigen for raising antibodies against the GPI-PLC and as a probe for interacting proteins by establishing that the anti-GPI-PLC antibody recognized the GPI-PLC alone when the enzyme, as an MBP fusion protein, was expressed in *E. coli*. This use and conclusion was verified in three steps: (1) by DNA sequencing of the expressed GPI-PLC gene to establish that the gene inserted did encode the GPI-PLC-MBP fusion protein, (2) that the immunoprecipitated protein from extracts of the recombinant *E. coli* was the GPI-PLC-MBP fusion protein when assess by MALDI-TOF MS and (3) that the antibody only recognized the GPI-PLC portion of the fusion protein when the fusion protein was cleaved at the TEV protease cleavage site between the two domains and, following separation both portions blotted with the antibody and then sequenced by MALDI-TOF MS.

The anti-GPI-PLC recognized the purified fusion protein as well as the GPI-PLC derived from the fusion protein after TEV protease cleavage (Supporting Information Fig. S1, panel A, image 2, tracks a and b respectively). The MBP, derived from the fusion protein after TEV cleavage, was not recognized by the anti-GPI-PLC (supporting information Fig. S1, panel A, image 2, track b). In a second experiment the band corresponding to the fusion protein and that corresponding to the GPI-PLC, after TEV cleavage of the fusion protein, were cut from the gel, eluted, trypsin cleaved and subjected to MALDI-TOF MS. In the case of the fusion protein, peptides corresponding to the GPI-PLC (50% sequence coverage) and the MBP (51% sequence coverage) were detected (supporting information Fig S1, panel B). In the case of the GPI-PLC protein band, derived by TEV cleavage, only peptides corresponding to the GPI-PLC were detected (supplementary material Fig. S1, panel C) with overall sequence coverage of 23%.

**Surface labelling of cells does not label internal tubulin**

Surface labelling of bloodstream forms of *T. brucei* was found not to label tubulin, which is an internal protein (Fig. S2).

**Calculation of the minimum detection level for the GPI-PLC if it were located in a variety of different cellular compartments compared to the level detected in the linear array on the flagellar array**

The minimum % of the GPI-PLC signal in the linear array that could be detected if it were uniformly dispersed in the plasma membrane was calculated to be 22.4 % using the relationship (Equation 1) immediately below:

minimum integrated projected area of

minimum % of GPI-PLC intensities detectable different site

signal in linear array = X X 100 Eq. 1

detectable elsewhere integrated pixel intensity projected area of the found under ideal conditions GPI-PLC linear array

where -

(1) minimum integrated pixel intensity measured = 427,312.

(2) integrated pixel intensity measured under ideal conditions = 7,226,942.

(3) projected area of the GPI-PLC linear array measured = 9.843 μm^2^.

(4) measured projected area of the plasma membrane as the different site = 37.2 μm^2^.

The minimum % of the GPI-PLC signal in the linear array that could be detected if it were uniformly dispersed in the glycosomes was calculated to be 2.4% using Equation 1 above and where the projected area of all of the glycosomes of 1 cell, required for this calculation, has itself been calculated to be 15.8 μm^2^, using Equation 2 below

calculated projected πr^2^ number of glycosomes cell volume in μm^3^

area of all of the = (true area 1 glycosome) X X X Eq 2

glycosomes in 1 cell 4πr^2^ μm^3^ 1 cell

and where -

(1) the true area of 1 glycosome taken from reference [36] = 0.233 μm^2^

(2) the number of glycosomes per unit volume of 1 μm^3^ taken from reference [36]

= 3.97

(3) the measured volume of 1 cell taken from reference [59] = 17.1 μm^3^

(4) the ratio of the projected area of a glycosome to that of the true area of the glycosome is the ratio of the area of a circle to that of a sphere of the same radius = πr^2^/4πr^2^ = ¼.

The minimum % of the GPI-PLC signal in the linear array that could be detected if it were uniformly dispersed in the flagellar pocket was calculated to be 1.00 %, again using Equation 1 above and where the measured projected area of the flagellar pocket = 1.67 + 0.51 μm^2^ (n=100) from our own unpublished data.

**Supporting Information Figure legends**

Figure S1. **Specificity of the anti-GPI-PLC antibody for the GPI-PLC within the recombinant MBP-GPI-PLC fusion protein.**

The purified maltose binding protein-GPI-PLC (MBP-GPI-PLC) fusion protein and the TEV cleaved purified MBP-GPI-PLC fusion protein was subjected to SDS-PAGE (panel A, image 1) and western blotting (panel A, image 2). In a second experiment, the band corresponding to the purified fusion protein and that corresponding to the GPI-PLC, derived by TEV cleavage of the recombinant immunoprecipitated fusion protein, were cut from the gel, eluted, trypsin cleaved and subjected to MALDI-TOF MS. Peptides corresponding to the GPI-PLC derived from the fusion protein are shown (single underlined) in panel B and gave 50 % sequence coverage of the GPI-PLC. Peptides corresponding to the maltose binding protein from the fusion protein are also shown (double underlined) in panel B and gave 51 % sequence coverage of the maltose binding protein. Peptides corresponding to the GPI-PLC, derived by TEV cleavage of the purified fusion protein, are shown in panel (C) and gave overall sequence coverage of 23 %.

Figure S2 **Immunoprecipitation of cytoplasmic tubulin in detergent lysates of surface labelled cells.**

Pleomorphic populations of ILTat 1.1 (5 x 10^7^ cells / ml) were surface biotinylated and the excess biotinylation reagent inactivated with 5 mM glycine, cells detergent-lysed, lysates centrifuged and the clear supernatants subjected to immunoprecipitation as described in Methods. Soluble tubulin was removed with anti-tubulin IgG bound to protein A-Sepharose beads and, following a wash, the immune complexes were removed by boiling for 2 min in SDS sample buffer and their constituent proteins separated by SDS-PAGE followed by Western blotting using (Panel A) anti-tubulin primary antibody followed by horseradish-conjugated secondary antibody or (Panel B) horseradish-conjugated streptavidin. In each case lane 1 contains the supernatant of a cell lysate (2 x 10^7^ cell equivalents); lane 2 contains the immunoprecipitated protein (2 x 10^8^ cell equivalents); lane 3 contains the supernatant of the immunoprecipitation reaction (2 x 10^8^ cell equivalents).
